# *Hostile environments?* Down’s syndrome and genetic screening in contemporary culture

**DOI:** 10.1136/medhum-2020-012066

**Published:** 2021-06-16

**Authors:** Lucy Burke

**Affiliations:** Department of English, Manchester Metropolitan University, Manchester, UK

**Keywords:** genetics, literature, medical humanities, reproductive medicine

## Abstract

This essay explores the complex entanglement of new reproductive technologies, genetics, health economics, rights-based discourses and ethical considerations of the value of human life with particular reference to representations of Down’s syndrome and the identification of trisomy 21. Prompted by the debates that have occurred in the wake of the adoption of non-invasive prenatal testing (NIPT), the essay considers the representation of Down’s syndrome and prenatal testing in bioethical discourse, feminist writings on reproductive autonomy and disability studies and in a work of popular fiction, Yrsa Sigurdardóttir’s *Someone To Look Over Me* (2013), a novel set in Iceland during the post-2008 financial crisis. It argues that the conjunction of neo-utilitarian and neoliberal and biomedical models produce a hostile environment in which the concrete particularities of disabled people’s lives and experiences are placed under erasure for a ‘genetic fiction’ that imagines the life of the ‘not yet born’ infant with Down’s syndrome as depleted, diminished and burdensome. With close reference to the depiction of Down’s syndrome and learning disability in the novel, my reading explores the ways in which the generic conventions of crime fiction intersect with ideas about economics, politics and learning disability, to mediate an exploration of human value and social justice that troubles dominant deficit-led constructions of disability.

## Introduction

This essay considers the ways in which Down’s syndrome (trisomy 21) is imagined and materialised in contemporary culture across different domains; within the discourses and apparatus of prenatal testing, recent feminist and disability scholarship, and in popular genre fiction, in this instance, the Icelandic author Yrsa Sigurdardóttir’s novel *Someone to Watch Over Me* (Sigurdardóttir 2013). It explores the complex entanglement of new reproductive technologies, genetics, health economics, rights-based discourses and ethical considerations of the value of human life, exploring the articulation of ableism with what Andre Gorz describes as a resolutely instrumental economic rationality “dominated by the concern for efficiency, productivity, and optimum performance”(Gorz 2012, 44). Setting out to identify some of the genetic fictions that have emerged as an effect of these relationships, the essay speaks to debates within and without feminism and disability studies as to the implications of new genetic technologies on the lives of disabled people, particularly the extent to which these technologies render some groups of people with genetic conditions existentially vulnerable, with the potential literally to screen them out of existence. It makes a case for the significance of imaginative literature in this context in enabling a more nuanced understanding of these complex debates via a critical reading of Sigurdardóttir’s novel that opens up a space for the expression of alternative conceptions of human value embodied in eccentric figures characterised by a strong difference; a young man with Down’s syndrome and a young autistic man.

Mindful of the contemporary political usage of the phrase ‘hostile environment’ in the UK to convey the creation of an environment in which the lives of migrants are deliberately rendered unbearably difficult (Liberty Human Rights 2019), I deploy it here to describe the emergence and embedding of thinking about prenatal genetic testing and Down’s syndrome across multiple, overlapping forms and sites of learning in which disability is predominantly framed in terms of deficit, difficulty, parental disappointment and struggle. This ‘pedagogy’ (Giroux 2004) of suffering and parental burden taps into the realities of systemic ableism, from lack of support, poverty, poor educational opportunities, low aspiration and unequal access to healthcare but does so in order to reinforce rather than challenge structural inequality, discriminatory attitudes and practices. As Gareth Thomas notes, screening for Down’s syndrome has become a routine element of prenatal care and this in itself enmeshes perceptions of what it means to identify trisomy 21 with ideas of risk and the potential (and pressure) to take ‘preventative’ action (Thomas 2017, 1).

The first part of this essay maps the contours of this hostile environment including justifications and challenges to prenatal screening programmes and the implications of the adoption of particular technologies most recently and notably that of cell-free DNA (cfDNA) testing, commonly known as non-invasive prenatal testing (NIPT). This technique, marketed globally under the brand name the Harmony, involves taking a blood sample from a pregnant person in the first trimester at 9 or 10 weeks and works by counting the number of placental cfDNA fragments from the different chromosomes present in the mother’s blood. It enables the measurement of the underlying genetic makeup of trisomy 21 (Down’s syndrome), trisomy 18 (Edwards syndrome) and trisomy 13 (Patau syndrome) (Nuffield Council on Bioethics 2017). The stated advantages of NIPT is that it is more accurate than the combined test (Taylor-Phillips et al. 2016) and carries no known risk of miscarriage (Public Health England Blog 2016). However, as I will discuss, this screening technique presents a significant ethical challenge from a disability rights-based perspective.

I go on to consider some of the material effects of this assemblage of techniques, epistemologies and bodies, focusing particularly on the evocation of two powerful genetic fictions in the fields of prenatal genetic testing and discourses of reproductive choice in contemporary feminism: the imagined but not yet/perhaps never to be born infant with Down’s syndrome and the imagined but not yet/perhaps never to be materialised figure of the ‘mother’ who suffers. I focus here on the place and function of trisomy 21 in prenatal diagnosis and argue that by virtue of the ease with which trisomy 21 is detected, Down’s syndrome has come to function as *the* imagined difference that encodes fears about the value and ‘quality’ of disabled peoples’ lives more generally. It thus carries the freight of a deeply entrenched ableism despite all that we know about the rich and varied lives that people with Down’s syndrome lead and indeed about contingency and the potential for any child or adult to develop an illness or impairment, what Marianne Hirsch describes as our “common vulnerability emerging from the condition of living in bodies and in time” (Hirsch 2016, 80).

I develop this argument fully cognizant of the tensions that often emerge between a feminist pro-choice perspective (a position with which I agree) and a disability rights-based argument that the detection of trisomy 21 alone is not sufficient grounds on which to terminate an otherwise wanted pregnancy (a position with which I also agree). However, it would be disingenuous to present this as an easy terrain to navigate in practice and it requires an acknowledgement of the profoundly ‘messy’, complicated and contextual nature of decision-making, particularly in an ableist, economically unequal culture dominated by neo-utilitarian premises about human value. Indebted to Alison Piepmeier’s (Piepmeier 2013) provocative critique of dominant feminist framings of prenatal testing and disability, my discussion of Ilana Löwy’s (Löwy 2017,^2018^) extensive and influential work on prenatal testing explores this problem with a particular focus on the assumptions about disability that underpin her analysis of reproductive choice including the absence of the voices of disabled people and the dismissal of the epistemic privilege experienced by the parents of disabled children (of which I am one) when this contradicts the stereotypes of “mothers as victims” and Downs’ syndrome as a “tragedy” (Piepmeier 2013, 165).

The final part of the essay turns to the popular genre of Nordic crime fiction in order to explore the articulation of ideas about Down’s syndrome, learning disability and parenting with a different epistemology of human value, thus giving rise to a different kind of ‘genetic fiction’ to that which I identify in work such as Löwy’s. The plot of Sigurdardóttir’s *Someone To Look Over Me* (2013) unfolds around the exoneration of a young man with Down’s syndrome who has been incarcerated for arson and the associated deaths of five people in the residential home in which he was placed. The novel is set in Iceland in the wake of the global financial crash of 2008, and weaves together a story about economic collapse, mendacity, deceit and the abuse of power at the centre of which are two learning disabled characters. My reading considers the ways in which ideas about art, disability, cognitive difference and marginality are integral to the novel’s critique of the economic and political culture that precipitated the financial crisis. I argue that Down’s syndrome and autism become the locus or expression of what Michel Foucault describes as the “insurrection of subjugated knowledges”(Foucault 1980, 81), emerging from the periphery to expose and trouble ableist and neoliberal assumptions about what and who matters, concluding with a discussion of an alternative conception of belonging, value and identity in Garland-Thomson (2011) idea of “misfitting”.

## Valuing people?

Much recent scholarship in reproductive ethics and public policy has focused on the financial, economic and societal impact of disability and therefore the economic benefits of prenatal genetic screening for readily detectible conditions such as Down’s syndrome. For instance, in their essay ‘The Economic Costs of Childhood Disability’, (Stabile and Allin 2012) set out to calculate the direct, indirect and long-term economic costs of having a disabled child in order to conclude “that many expensive interventions to prevent and reduce childhood disability might well be justified by a cost-benefit calculation” (Stabile and Allin 2012, 65). Drawing on the work of prominent neoliberal thinkers such as Gary Becker and the health economist, Michael Grossman, the authors embrace a model of health as a “stock”, an “input into the production of human capital, the development of the competencies and knowledge that increase one’s ability to work and be productive” (Stabile and Allin 2012, 66). Like so much work in the field of health economics in the global north, there is no endeavour to situate the calculation of the financial costs associated with disability alongside any sustained ethical consideration of the value of human life in all its diversity or even any acknowledgement of the ethical questions that circumscribe their approach. Their analysis draws on and reinforces a deficit model of disability in which the disabled child is figured *only* as the locus of parental suffering and strain, financial and societal burden. Abstracted and hypothetical, this pleasure stealing, resource consuming, energy sapping figure is the absent centre around which various forms of loss and detriment are traced. The uniqueness and concrete particularly of life—*a* life—is placed in parenthesis for a calculation of human value reduced to a stark economic logic and a narrowly conceived notion of productivity and societal contribution.

It is on this terrain that health economists working in advanced economies have produced cost/benefit evaluations of different screening technologies, for instance, Song *et al* compare the costs of early detection of trisomy 21 and termination with the costs of the first 5 years of life for a person with Down’s syndrome, noting that “clinical benefits are realized in the setting of also achieving cost savings” (Song, Musci, and Caughey 2013, 1185). This logic characterises numerous papers that assess the economic advantages in particular national healthcare contexts of entering into a commercial relationship with the laboratories that produce and market the technology (Beulen et al. 2014; Hui and Hyett 2013; Neyt, Hulstaert, and Gyselaers 2014). As Kibel and Vanstone note, “Cost-effectiveness evaluations of NIPT often assess NIPT’s ability to deliver on goals (ie, preventing the birth of children with disabilities) that social and ethical analyses suggest it should not have” (Kibel and Vanstone 2017).

Indeed, reflecting on the ethical dilemmas to which the widespread adoption of technologies such as NIPT give rise, Megan Best has noted that the routinisation of prenatal genetic screening often occurs without the comprehensive counselling that accompanies other kinds of genetic testing and on the basis of an “assumed consent” rather than “true individual preference for fetal chromosomal information” (Best 2018, 115). She argues that “medical counselling is often construed as a medical directive by the women seeking antenatal care” on the basis of “an innate power imbalance in the doctor-patient relationship which puts the woman in a position where the autonomous choice to screen is in fact experienced as an inability to justify not doing so” (Best 2018, 116). In other words, it is very difficult to opt out of this kind of testing regime, particularly when it is presented as being in everyone’s best interest. If this highlights the difficulties that attend the realisation of informed consent in prenatal screening programmes, research also indicates that the very perception and representation of NIPT as ‘easy or just another blood test’ also serves as a barrier to informed decision-making (Cernat et al. 2019, 2) because people are not necessarily aware of or prepared for the potential outcome of the test, although there is often an assumption that the identification of a genetic condition such as Down’s syndrome will end in the termination of the pregnancy.

When we consider questions of choice and risk in this context, it is also important to remember the commercial interests that drive the roll out of technologies such as NIPT and the ways in which these contribute to the creation of a culture in which selective abortion is routinised as an effect of the testing regime and consequently negative perceptions of particular conditions. As Piepmeier notes, “having the test isn’t a neutral situation; it can create and contribute to fear” (Piepmeier 2013, 166). For instance, NIPT is currently marketed by private health providers as a technique to identify a number of other, often very rare, genetic conditions including those caused by unusual numbers of the sex (X and Y) chromosomes, such as Turner syndrome and triple X syndrome, and those caused by small bits of DNA missing, called microdeletions, such as Prader-Willi syndrome and 5p deletion syndrome. Research indicates that NIPT is not particularly effective or accurate in identifying these conditions and the Nuffield Council on Bioethics in the UK has voiced concerns about the tendency to present false positives in this context (Nuffield Council on Bioethics blog 2019; Taylor-Phillips et al. 2016). Marketing that claims 99% accuracy thus exploits and profits on understandings of risk and associated anxieties. This commercial logic emphasises the degree to which the concept of ‘choice’ must be situated in a critical analysis of the economic drivers that underpin the biopolitical logic of the prenatal diagnosis (PND) apparatus.

## Screening for trisomy 21: Down’s syndrome as the paradigmatic positive result of PND

As outlined above, the centrality of ideas of disability as a cost (emotional, familial, financial, societal) to the ideological justification and implementation of screening programmes is an important reminder of the systemic ableism that has long characterised healthcare as a biopolitical apparatus, the provision of which is often shaped by neoliberal and neo-utilitarian principles rather than the endeavour fully to recognise the value of human diversity. Indeed, if we consider the history of prenatal genetic screening and diagnosis in more detail, it is evident that the articulation of utilitarianism and ableism is deeply imbricated in the development of this area of genomic medicine.

Prenatal diagnosis or PND refers to the “scrutiny of the fetus coupled with the option to terminate pregnancy” (Löwy 2018) and was established as a routine element of pregnancy care in the late 1960s and early 1970s. Its implementation was the product, as medical anthropologist Ilana Löwy notes, of “four distinct developments in biomedicine—the perfection of amniocentesis, the rise of cytogenetics (the genetic study of cells), the application of new biochemical approaches to the study of amniotic fluid and pregnant women’s serum, and the development of obstetrical ultrasound”—along with the decriminalisation of abortion in some countries (Löwy 2018, 3). The identification of a fetus with trisomy 21 is, as Löwy notes, “the paradigmatic positive result of PND” and the paradigmatic justification for the termination of pregnancy (Löwy 2018, 220). Löwy cites Susan Lindee’s assertion that “the selective abortion of affected fetuses was and remains the primary intervention of genomic medicine” noting that this rationale is embedded in the 20th century history of clinical genetics (Lindee 2002; Löwy 2018, 5) and manifest in the concerns of early genetic researchers such as Julia Bell (the scientist who identified Fragile X). In a correspondence in the *British Medical Journal*, Bell focused on three main concerns:

1. The risks of severe handicap to the unborn child.
2. The risks of acute distress and difficulty for the potential parent, perhaps for the rest of her life.
3. The burden likely to rest on the Welfare state (Bell 1959, 1302; Löwy 2018, 203).

Bell’s reference to “the burden likely to rest upon the Welfare state” demonstrates the degree to which the history of genomic discourse is entangled with biopolitical and economic considerations that subject the value of some lives to the instrumentalist logic of the cost/benefit analysis based on perceptions of societal burden. Indeed, the development of prenatal testing and the establishment of health economics as a subdiscipline of economics emerge in the same period. Löwy notes that “in discussing the decision to introduce screening for Down’s syndrome in France, a French public health expert explains that politicians could not openly admit that their aim was to reduce the number of children born with chromosomal anomalies in order to decrease the costs of care for these children” (Löwy 2018, 213). The barely concealed hostility to “disability rights activists” that surfaces throughout Löwy’s work is underpinned by a similar economic logic, noting that the decision to have an “impaired child”runs the risk of “depleting the family’s financial and emotional resources” in a context of “uncertainty about the fate of disabled people in economically and politically unstable times” (Löwy 2018, 214):

Few people object to the principle that society should provide sufficient help to disabled persons and their families. But acceptance of the generous principle is hampered by practical difficulties in fulfilling all the urgent and often competing societal needs (Löwy 2018, 161).

Löwy’s position here brings together a feminist pro-choice perspective with a neo-utilitarian and deficit led view of disability; the ‘impaired child’ is associated with depletion, insecurity and unwarranted burden. In this respect, her response is emblematic of the resignification of liberationist themes from feminism in the era of globalisation and financial crisis, and of a version of feminism that happily coexists with ableism. Löwy presents an argument for gender equality predicated on reproductive autonomy that goes hand in hand with an ableist justification of political and ethical inequality ideologically legitimated in overtly economic terms. In Löwy’s words, prenatal diagnosis is useful as a “gendered risk management technology” (Löwy 2018, 15) in which the pregnant person essentially either chooses to assert themselves and their autonomy or consent to their own subjugation to the insatiable wants and needs of their disabled child, a decision that is implicitly framed as selfish act in light of “all the urgent and competing societal needs”.

This kind of emphasis and understanding of ‘choice’ and autonomy underpins a number of feminist writings on reproductive rights (Rothman 1993; Bender and de Gramont 2010) and, as Alison Kafer puts it, makes “disability do the work of defending abortion” on the basis of profoundly discriminatory stereotypes about disabled people’s lives and experiences of parenting disabled children (Kafer 2013, 167). The notion of ‘risk management’ in work such as Löwy’s is framed by a stark economic rationality and an attendant conception of disability as parasitical dependency and familial burden. There is little consideration of “messier questions and concerns” (Piepmeier 2013, 176) and the intimate, familial, communal and socioeconomic contexts that frame decision-making and delimit the idea of ‘choice’ as some unmediated expression of individual volition. As Piepmeier suggests, shifting the focus from an individualised framework to one based on the principle of reproductive justice requires a response that attends to and recognises the ‘humanity’ of disabled people and the relationships between human rights and economic justice in order to “create communities that make decisions possible” (Piepmeier 2013, 182).

### The expressivist objection

The longstanding disquiet about the implications of prenatal genetic testing for many disability scholars and activists is known as the expressivist objection. This argument claims that to eliminate a particular genetic trait through selective abortion “expresses (and presupposes) negative, extremely damaging judgements about the value of disabled persons” (Edwards 2004; Gonter 2004). In an oft-cited passage from *The Rejected Body* (1996), the feminist philosopher Susan Wendell argues that:

the widespread use of selective abortion to reduce the number of people born with disabilities … sends a message to children and adults with disabilities, especially people who have genetic or prenatal disabilities, that “we do not want any more like you” (Wendell 1996, 153).

In a similar vein, Adrienne Asch argues that prenatal testing repeats and reinforces the same tendency towards letting the part, that is, a single trait, stand in for the whole; a metonymic impulse that characterises discriminatory attitudes towards disabled people more generally (Asch in Parens and Asch 1999, S2). In other words, it reproduces the tendency to reduce and flatten disabled peoples’ lives to the contours of a specific impairment or diagnostic category as if a particular label ‘unlocks’ or explains everything about a disabled person, or all a non-disabled person might need to know.

This expressivist objection to prenatal testing has been characterised as ‘avoiding difficult questions’ by scholars such as Löwy and as theoretically incoherent by bioethicists such as John Harris (John 2000). Harris, who is unapologetic in his conviction that “people should practise eugenics, if by that is understood the attempt to produce healthy, non-disabled children” (John 2000, 99) also argues that “deliberately to make a reproductive choice knowing that the resulting child will be significantly disabled is morally problematic, and often morally wrong” (John 2000, 96). Likewise, Richard Dawkins has argued that there is a ‘moral’ imperative’ to terminate a pregnancy if Down’s syndrome is detected on the basis of an impressionistic and unevidenced claim that the birth of a disabled infant increases the amount of suffering in the world (Dawkins 2014). Others such as Daniel Brock have made similar arguments to Harris and Dawkins but have endeavoured to do so in response to aspects of the expressivist objection in a manner which, as Eva Kittay brilliantly demonstrates, is often opaque and rather incoherent itself (Brock 1995, 2004, 2005).

As Kittay argues, the most notable characteristic of these attempts “to secure a moral requirement to select against disability, while wishing to avoid denigrating disabled people” is that they remain wedded to a deficit-led conception of disability as “only the occasion for suffering” (Kittay 2017, 185). In other words, they unfold around a vision of disabled life as worse than no life at all and argue that to entertain the realisation of such a life is to affect harm in the “creation of a world with less opportunity”or “diminished”opportunity (Kittay 2017, 189). As Alison Kafer notes, “if disability is conceptualised as a terrible unending tragedy, then any future that includes disability can only be a future to avoid […] the value of a disability-free future is seen as self-evident” (Kafer 2013, 2) As Kafer’s work emphasises, these arguments in which screening out disability is justified as a ‘moral imperative’ depend on an entirely abstract projection, an “imagined future” (Kafer 2013, 2) and therefore an essentially fictional construct of disability, cut adrift from the concrete particularities that might give it any substantive meaning. As Kittay notes,

Life is so strewn with contingencies that the presence or absence of a disability in an individual’s life is still a poor predictor of what would be a better life for that person. The child who is without significant impairments may be born into a family that is not quite as loving, not quite as resourceful, not quite as accepting, as a person with severe impairments. A significantly impaired child blessed with loving parents, and a supportive environment, and undaunted by the challenges of her impairment, may flourish and have a wonderful life. […] The ceteris paribus clause leaves us with an empty abstraction, an idealized condition with little relevance to our nonidealized world. It cannot support the claim that a disabled life, all things considered, is a worse life than an able one (Kittay 2017, 187).

Any engagement with lived experience or indeed any contextualised comparison of moments of pleasure, enjoyment, loss and pain in disabled and non-disabled lives would complicate and disturb the singular, condemnatory logic of this kind of ethical position in which the potential harms of an unlived life are imagined and flow from a fundamentally impoverished and ableist view of disability. It is notable that the perspectives of people with learning disabilities such as Down’s syndrome are rarely included in these bioethical pronouncements, although in their recent consultation about the adoption of NIPT in the UK, the Nuffield Council on Bioethics managed to record the views of six people who identified with this label (Barter 2017).

However, it is not simply the imagined suffering of the not yet born disabled child but also crucially the imagined suffering of the family of that child that comes into play in these debates. In her work on prenatal testing, Löwy, for instance, is scathing in her condemnation of unnamed ‘disability activists’ for suggesting that there are “substantial gratifications and unexpected joys”associated with raising what she describes as “a special-needs child” (Löwy 2018, 219). “It is difficult to achieve credibility”, she notes, “through the telling of partial truths” (Löwy 2018, 220). As this suggests, it is difficult for her to imagine that the life of someone with a child with a learning disability could be anything other than abject and unalloyed misery and this, in turn, produces a constitutive suspicion of any argument to the contrary. Löwy resists any acknowledgement of the epistemic privilege that being the parent of a disabled child confers but instead reflects this back as either ‘over-compensation’ or self-deception (the ‘telling of partial truths’), invalidating personal testimony on the basis that it cannot possibly be ‘true’ if it does not conform to the abstracted deficit model from which her analysis proceeds.

### Genetic fictions and the significance of the literary

My aim in the sections above is to highlight the function and centrality of Down’s syndrome to discussions about prenatal genetic testing, the intersection of economic and medical discourses and to identify the main characters or subjects that that populate dominant discourses around screening practices; the yet to be born infant with Down’s syndrome and the potential yet to be materialised parent of that child. Crucially, we need to recognise that these imagined or projected subjects are distinct effects of this apparatus. They are genetic fictions so to speak that express, as Piepmeier notes, “a culture with skewed, dehumanising views of disability” (Piepmeier 2013, 163) in which Down’s syndrome and being the parent of a child with Down’s syndrome are often freighted with a raft of negative attributes, casting a disabled life as intrinsically diminished and burdensome. We might describe this assemblage of practices, ideas and pressures as producing a hostile environment because there is very little space here afforded to disabled people’s voices or the concrete particularities of lived experience in all its variability. Instead, these spectral projections function as empty placeholders, harbingers of tragedy, depletion, deficit and struggle. These “grim imagined futures”, as Kafer puts it (Kafer 2013, 2), fail to capture the diverse realities of people’s lives and relationships and often compel disabled people and their families to justify their very existence and the quality and value of their lives in order to provide a counternarrative, a pressure to justify life itself rarely, if ever, experienced by non-disabled people.

I want to turn now to a consideration of the representation of Down’s syndrome, learning disability and parenthood in the popular genre of Nordic crime writing in order to explore the ways in a piece of genre fiction, specifically Icelandic author Yrsa Sigurdartottir’s novel *Someone to Watch over me* (2013), both engages with and troubles the kind of ableist, neoliberal and neo-utilitarian constructions of human value that I have been exploring and which underpin the place of trisomy 21 in the apparatus of prenatal genetic testing and its ideological centrality to the idea that some lives are disposable, not worth living or to be prevented for the benefit of the parent, family or society. My reading focuses on the ways in which the novel opens up a space for the expression of alternative conceptions of what and who matters within a broader consideration of the relationship between the law, social and economic justice and the activities of the state.

My decision to write about an Icelandic novel in this context is worth briefly reflecting on. In 2017, various global news reports indicated that Iceland was on the brink of becoming the first country to ‘eliminate’ Down’s syndrome, prompting renewed debate about the potential ‘screening out’ of particular groups of people and the emergence and cultural acceptance of a ‘new’ eugenics. Although these reports about the eradication of Down’s syndrome in Iceland have been disputed, not least by the Icelandic government, screening for chromosomal conditions is a well-established element of prenatal care (although NIPT is not currently offered), and over the last 10 years, only two to three children with Down’s syndrome have been born each year, making Siggurdartottir’s decision to write about Down’s syndrome in her novel, significant (Government of Iceland 2018). Iceland also has a distinct—possibly unique—national experience in relation to the interactions between the state and a commercial biotech DeCODE genetics/Amgen, with its aspirations to capitalise on large-scale whole-genome sequencing of the Icelandic population (Fortun 2008; Gudbjartsson et al. 2015). The DeCODE project was initially framed and marketed in the 1990s in relation to the perceived homogeneity of the population and the genealogy of the nation reaching centuries back in time, evoking images of an unbroken lineage of Viking warriors and notions of racial purity (Burke 2012, 200). Ethical debates around emergent genetic technologies and the implications of commercial relationships between the state, citizens and profit-making corporations are therefore culturally prominent and the subject of sustained reflection in novels such as Indridason (2006) *Tainted Blood*. Finally, the financial crisis of 2008 and subsequent recession in Iceland had major political ramifications and exposing the limitations and destructiveness of financial deregulation and neoliberal economics.

These concrete cultural determinants coalesce in the novel’s articulation of the relationships between the marginalised and the powerful in the context of a post-crash society enabling a textual reframing of the dominant representations of Down’s syndrome and parental burden that I have identified in the apparatus of prenatal testing and indeed, particular, non-intersectional, feminist writings on reproductive autonomy. Whereas the latter practices are often predicated on the erasure of the disabled subject as a someone with significance beyond attributions of suffering and burden, my reading illuminates the way in which the novel endows its least powerful characters with an epistemic privilege that exposes the systemic corruption, exploitation and greed that precipitated the global financial crisis, and which is predicated on a narrowly conceived and constitutively ableist conception of human value. In making this argument, I am also making a claim for the value of the literary and literary critical practices as equally important fields for exploring the ethical and political questions raised by genetic screening practices, health economics and feminist considerations of reproductive autonomy and intersectionality. As Mikhail Bakhtin argues, we can locate the ethical and political significance of novelistic discourse in its formal capacity to dialogise and defamiliarise different and competing social discourses, drawing our attention to asymmetrical distributions of power and authority (Bakhtin 2010, 262). What this means is that novelistic discourse offers a distinctive space wherein we encounter plural and often antagonistic discourses from different spheres, for instance, the political, the legal, the scientific and the technical playing out their contradictions through the stories of particular individuals and collectives facing particular sets of circumstances and challenges. Unlike the ‘genetic fictions’, we encounter in the discourses and practices of medicine or health economics then, my argument is that *Someone to Watch Over Me* presents a more capacious and generous space in which eccentric or marginal voices can break through the dominant and often exclusionary frameworks of the hostile environment faced by disabled people today.

### Subjugated knowledges and misfitting: *Someone to Watch Over Me*

*Someone To Watch Over Me* can be described as an allegory of the failure of the state to protect or ‘watch over’ its citizens in the wake of the rapid expansion and subsequent collapse of the Icelandic banking system. The narrative unfolds in the context of the severe financial crisis in Iceland following the global crash of 2008 in which overly enthusiastic Icelandic bankers played a small but significant role. The conventional ‘who done it’ of crime fiction is conjoined in the text with a ‘who didn’t do it’. The lawyer Thora Gudmundsdottir is employed to establish the innocence of Jakob, a young man with Down’s syndrome held in a secure psychiatric unit at Sogn for arson and the killing of five people in the residential care home in which he was placed against his will. Thora receives her instructions and payment from Josteinn Karlsson, a convicted paedophile serving an indefinite sentence in the same psychiatric unit on the grounds of “acute schizophrenia and other personality disorders” (17). The quest to exonerate Jakob takes place between January and March 2010 and traverses the key institutional spaces of biopolitical governmentality: the hospital, the prison, the Regional Office for the Disabled, the Ministry of Justice.

That the psychopathic Josteinn is the catalyst for Thora’s endeavour is indicative of a constitutive suspiciousness or sense of unease that pervades the story and threatens to destabilise the realist conventions of the crime genre itself. This unease is played out in frequent references to foul smells, breathing difficulties, panic and anxiety, suggesting something rotten at the heart of the society the novel describes. This is particularly evident in the Gothic undertow of the subnarrative that acts as a framing device for the main narrative about Jakob. This narrative describes the apparent ‘haunting’ of a family whose babysitter was killed in a hit and run accident on the way to look after their young son. The relationship of this framing narrative to Jakob’s story is unclear until the very end of the novel when the identity of the hit and run driver is revealed. The driver turns out to be Fanndis, the wife of Einvardur, a high-ranking official in the Ministry of Justice and mother of Tryggvi, a young man with autism who later dies in the fire at the care home for which Jakob is held responsible. What is ultimately revealed in the narrative then is a cover up and an act of injustice towards Jakob that originates at the very heart of the state, rather than as is first suggested in the apparent deviance of a marginalised individual with Down’s syndrome.

The generic and thematic disturbance encapsulated in the gothic elements of the text is bound up with a sense of profound distrust in individuals and institutions as a consequence of multiple and intersecting forms of risky behaviour. These behaviours include the failure to install a sprinkler system in the rapidly constructed ‘state of the art’ residential home in which Jakob is forced to live, the failure of the night staff in the home to protect its inhabitants from harm, including the rape of a young woman in a coma, and the use of the residents’ intravenous drips and oxygen in the sale of hangover treatments for young people in the vicinity. Ari, the original and corrupt lawyer assigned both to Josteinn and Jakob’s case is a gambling addict who throws out “his bills unopened” and fails in his professional obligation to represent his clients’ best interests, and Einvardur, a senior figure in the Ministry of Justice, is ultimately revealed to be complicit in the cover up of his wife’s role in the hit and run accident. The novel is thus populated with figures who behave irresponsibly, violating professional codes and ethical norms while seeking to conceal their culpability by assigning blame to Jakob.

The weakening of trust in the institutions of the state is most powerfully expressed in the symbolic alignment of the paedophile Josteinn and Ministry of Justice official Einvardur. These figures superficially at least are presented in opposition to one another. The recipient of ‘justice’, Josteinn, is described as physically abhorrent with thinning dark, slicked back hair, “yellow teeth” and “sour-smelling breath” that makes Thora recoil (128). In contrast, Einvardur Tryggvason, the agent of justice, is ‘spotless’:

His dark elegant suit appeared to shine and it was as if he’d just got up from the barber’s chair after a haircut and a close shave. His smile revealed white teeth that weren’t completely straight, but which gave him a character that defined the difference between a good-looking real person and a model. Strange as it might have seemed, it was precisely this imperfection that made him appear perfect (145).

Einvardur is initially approached by Thora (the lawyer) because Einvardur’s ‘severely autistic’ son, Tryggvi, died in the fire at the care home. We learn that prior to his death, Tryggvi was the recipient of an ‘unorthodox’ therapy that, we discover later, enables him to produce drawings of the hit and run accident. His endeavour to represent this traumatic experience and his new communicative intent causes his family rapidly to bring his therapy to an end in order to prevent him from revealing his mother’s culpability for the hit and run. The conviction of Jakob, orchestrated by Einvardur, is also used to divert attention from the family. This includes covering up the role of Einvardur’s daughter, Lena, in the fire itself. Einvardur’s ‘spotlessness’ and preparedness to sacrifice Jakob is symbolically aligned with Josteinn’s pathological inability to recognise or empathise with his victims. The use of ‘appearance’ in these descriptions is significant then in that it gestures towards the essentially untrustworthy nature of Einvardur as a figure and by extension the fundamental untrustworthiness of the apparatus of justice itself. The novel, in this sense, encapsulates the emergence of a new ‘structure of feeling’ (Williams 1977, 132) predicated on loss of faith in traditional state institutions and practices via a depiction of these key ‘actors’ as intrinsically unprincipled and self-serving.

Having mapped out the main elements of the narrative, I want to argue that the centrality of figures with learning disabilities in the novel is not incidental but integral to its engagement with questions of truth and justice. Thora’s quest unfolds as a well-meaning and soul-searching liberal education in the genetic diagnosis, health concerns and symptomatic characteristics of Down’s syndrome and autism and culminates in her acceptance of the value of Jakob’s life. We see her reflect on Jakob’s behaviour and looks, the decisions that Jakob’s mother has made and what it means to love a disabled child. If these reflections are somewhat stock responses to disability and Downs’ syndrome, what makes the novel interesting is the revelation that Jakob and Tryggvi are the repositories of ‘truth’ from the outset, although the ways in which they reveal the criminal acts that they have witnessed are not initially understood. Structurally speaking then, the revelation of ‘truth’ and justice is located not at the centre but at the periphery in the discourse of these marginal figures of disposable life. Jakob, in particular, is arguably emblematic of the broader vulnerability of citizens in the current post-2008 conjuncture. To maintain fictions of autonomy and choice under conditions of economic crisis here means that certain lives have to be sacrificed both literally and politically in order to protect those in a position of power. As the novel reveals, Fanndis’ guilt is concealed by the sacrifice of her son, Tryggvi, and the decision to silence him through the termination of his therapy. She is further protected by the indefinite incarceration of Jakob on the grounds of mental capacity. The assumption that Jakob and Tryggvi are disposable and unworthy of equal recognition and protection is ultimately challenged in the text in order to illuminate the corruption and inequality at the heart of a legal and political system that fails to value their lives.

Michel Foucault’s elaboration of the concept of “subjugated knowledges” is helpful here in making sense of the significance of Jakob and Tryggvi to the revelation to truth in the novel. Foucault points to “historical contents that have been buried or masked in functional coherences or formal systemizations […] a whole series of knowledges that have been disqualified as non-conceptual knowledges, as insufficiently elaborated knowledges: naive knowledges, hierarchically inferior knowledges, knowledges that are below the required level of erudition or scientificity” (Foucault 1980, 81). What we see in Tryggvi’s drawings and Jakob’s testimony is precisely the expression of ‘non-conceptual and insufficiently elaborated knowledges’ whose very emergence exposes the ethical limitations and brutality of the dominant epistemological frameworks that systematically disqualify or mask them.

I want to return then with this in mind to my earlier discussion of Löwy’s work and the apparatus of prenatal screening. Even the briefest of engagements with debates in health economics, bioethics and the work of scholars such as Löwy, indicate that the values of autonomy, choice, recognition and risk management are increasingly bound up with the exclusion of particular subjects, particularly those whose strong difference challenges deeply entrenched ableist norms. However, as this discussion indicates, arguments that support the principle of screening out Down’s syndrome as either a ‘moral imperative’ or a rational choice in a time of austerity do so via the repetition and reiteration of a decontextualised and abstracted idea—or genetic fiction—about what that label means, projecting meaning onto lives that have not yet been lived. This is a promissory discourse that unfolds around the assumption that a life with Down’s syndrome is essentially a life not worth living and it operates on a discursive level itself to screen out any recognition of the diverse, shifting and myriad experiences that make up any life. It is precisely this ableist premise with which Siggurdartottir’s novel opens but which its narrative comes to dismantle. In the novel, the structural position of Tryggvi and Jakob gives rise to an epistemic privilege that serves to trouble normative and ableist conceptions of value, and about what and who matters, opening up a space in which the revelation of truth and the realisation of justice are located in the most socially marginal figures.

It is here—in conclusion and in defence of lives easily construed as unworthy of living—that I want to introduce Rosemarie Garland-Thomson’s concept of misfitting. The term describes a critical practice that acknowledges the complex and material particularities of disabled peoples’ lives and the capacity to produce subjugated knowledges from which “an oppositional consciousness and politicised identity might arise” (Garland-Thomson 2011, 598). Fitting as she notes,

…is a comfortable and unremarkable majority experience of material anonymity … when we fit harmoniously into the world, we forget the truth of contingency because the world sustains us. When we experience misfitting, we recognise that disjuncture for its political potential, we expose the relational component and the fragility of fitting. Any of us can fit here today and misfit there tomorrow (Garland-Thomson 2011, 598).

In the current discussions of NIPT and prenatal diagnosis, Down’s syndrome operates as a ‘master signifier’ of disability, the paradigmatic difference to be identified, and the risk to be managed and, in the majority of cases, screened out. What is so important about Garland-Thomson’s argument is its emphasis on the complex particularities of people’s lives and the fragility and transience of ‘fitting’ itself. Such a position rejects the abstracted genetic fictions that emerge in the pedagogy of parental burden and discourse of ethical ‘harm’. Instead, this prompts us to recognise that it is not possible to eliminate human vulnerability or contingency, to accept that ‘misfitting’ is something anyone can experience at any point. Yet as Garland-Thomson notes, and as I tried to bring out in my reading of the novel, this very fact has the capacity to produce an oppositional consciousness; to reimagine the world in a way that is attentive and open to the varied particularities of disabled lives, an openness that goes beyond the reductive ableist prisms of tragedy and burden that predominate in biomedical and even certain strands of feminist thinking.

## Data Availability

Data sharing not applicable as no datasets generated and/or analysed for this study. No datasets generated or analysed.
